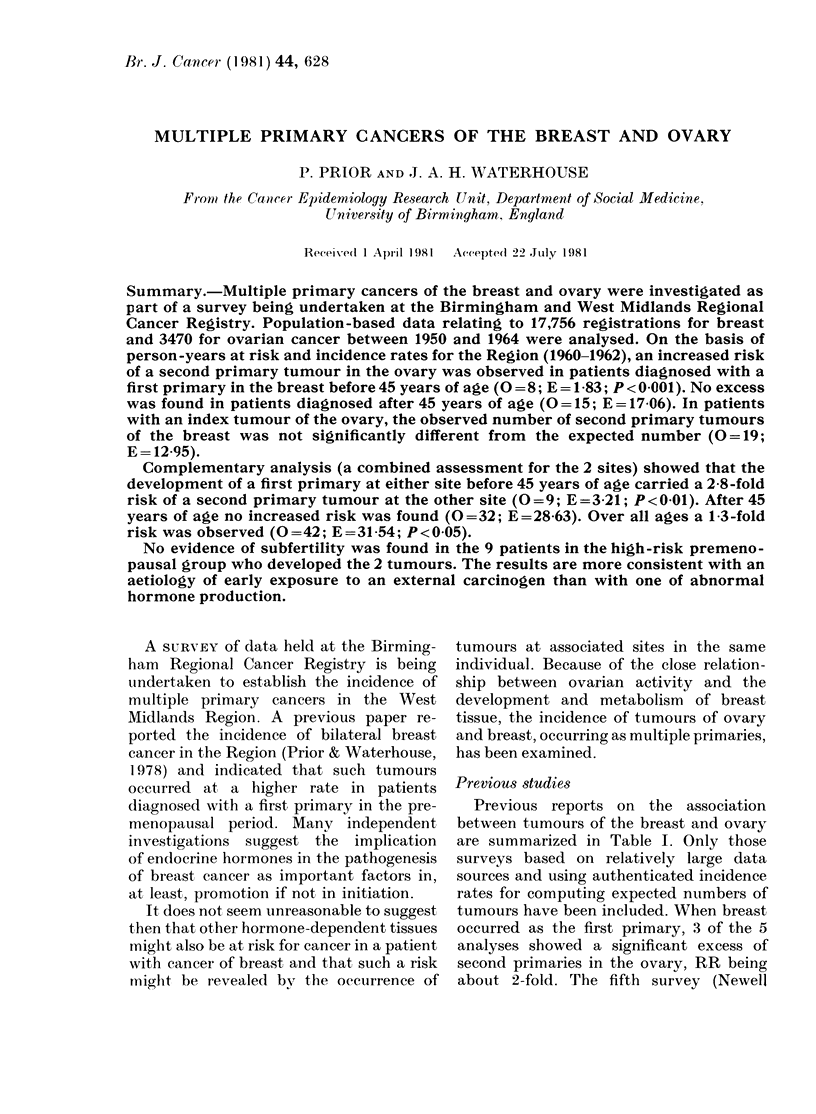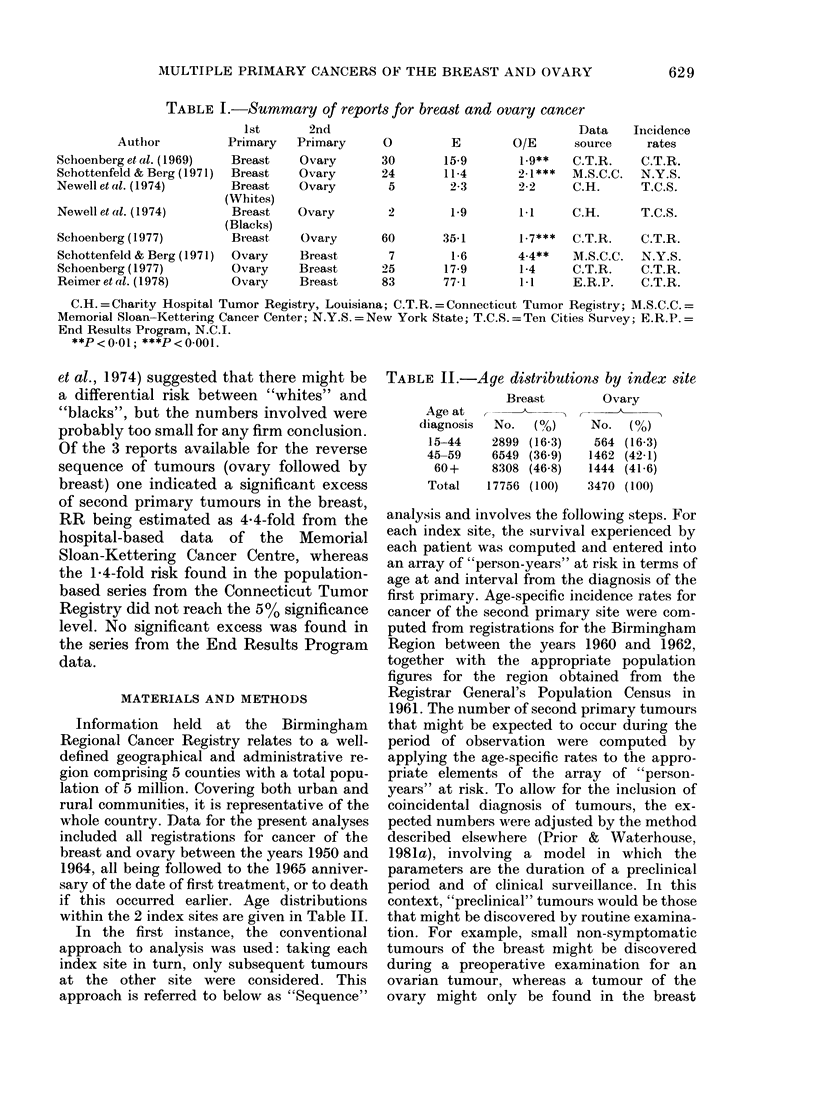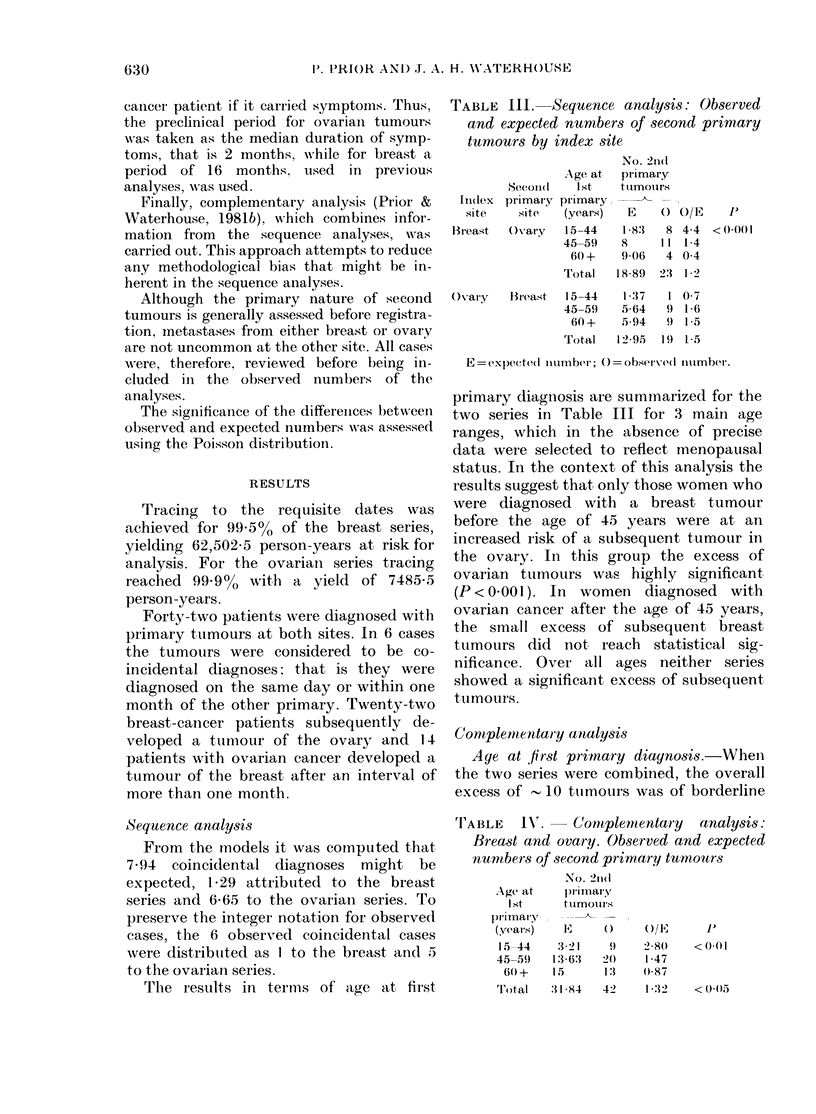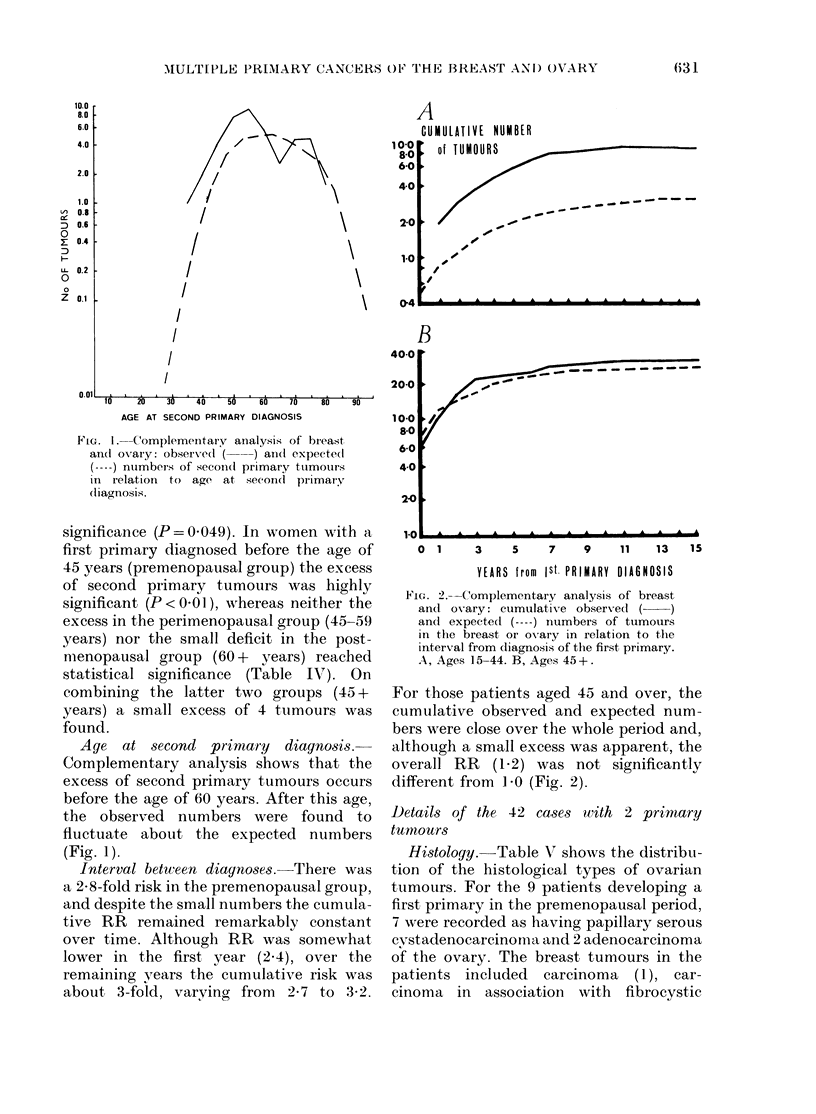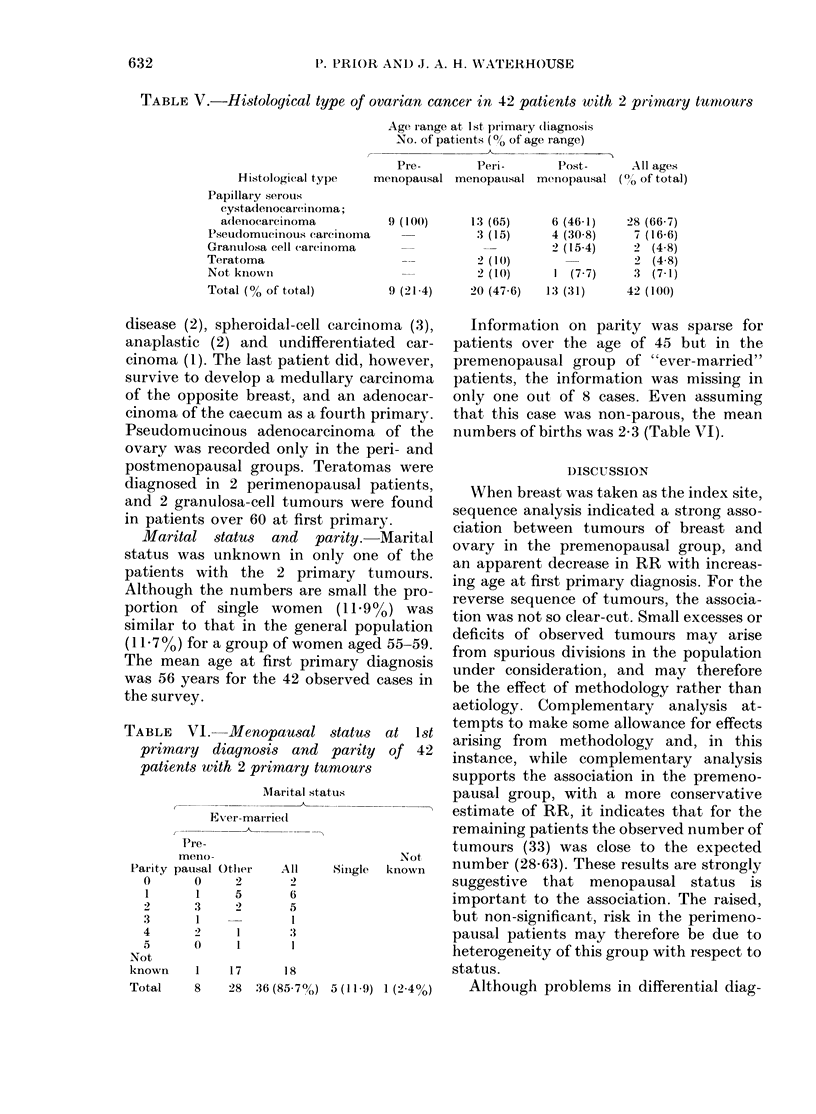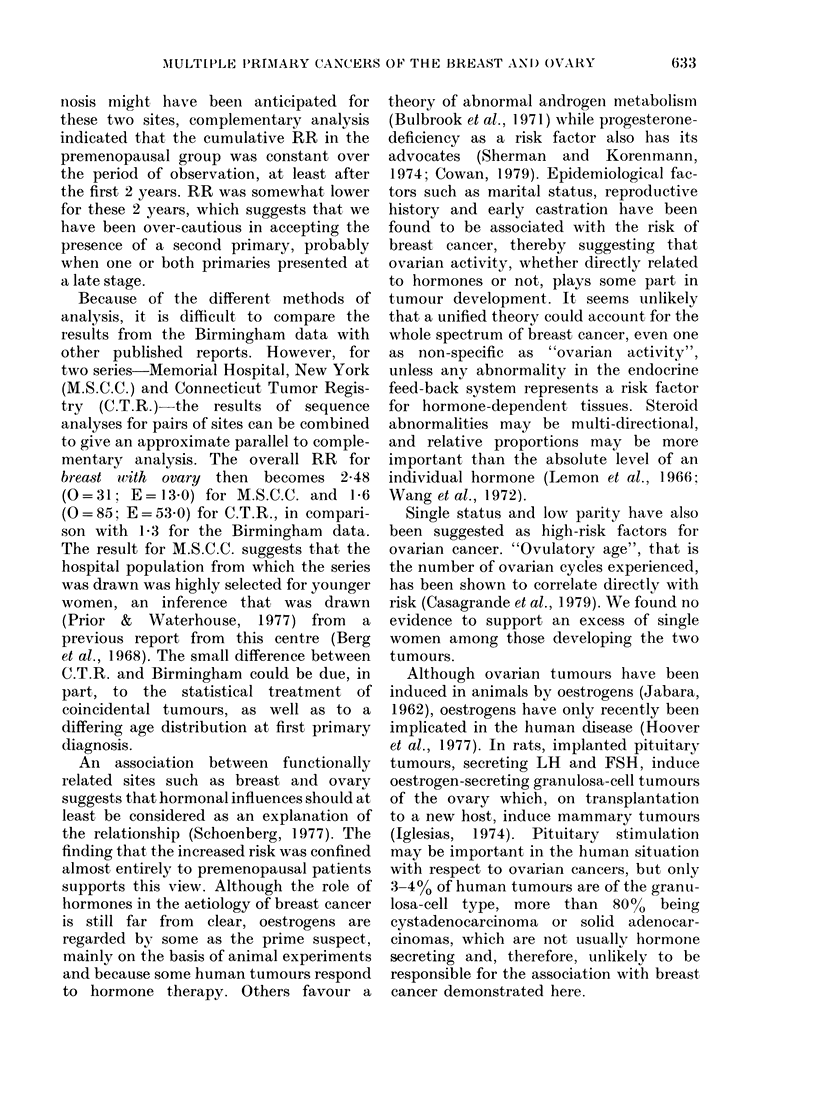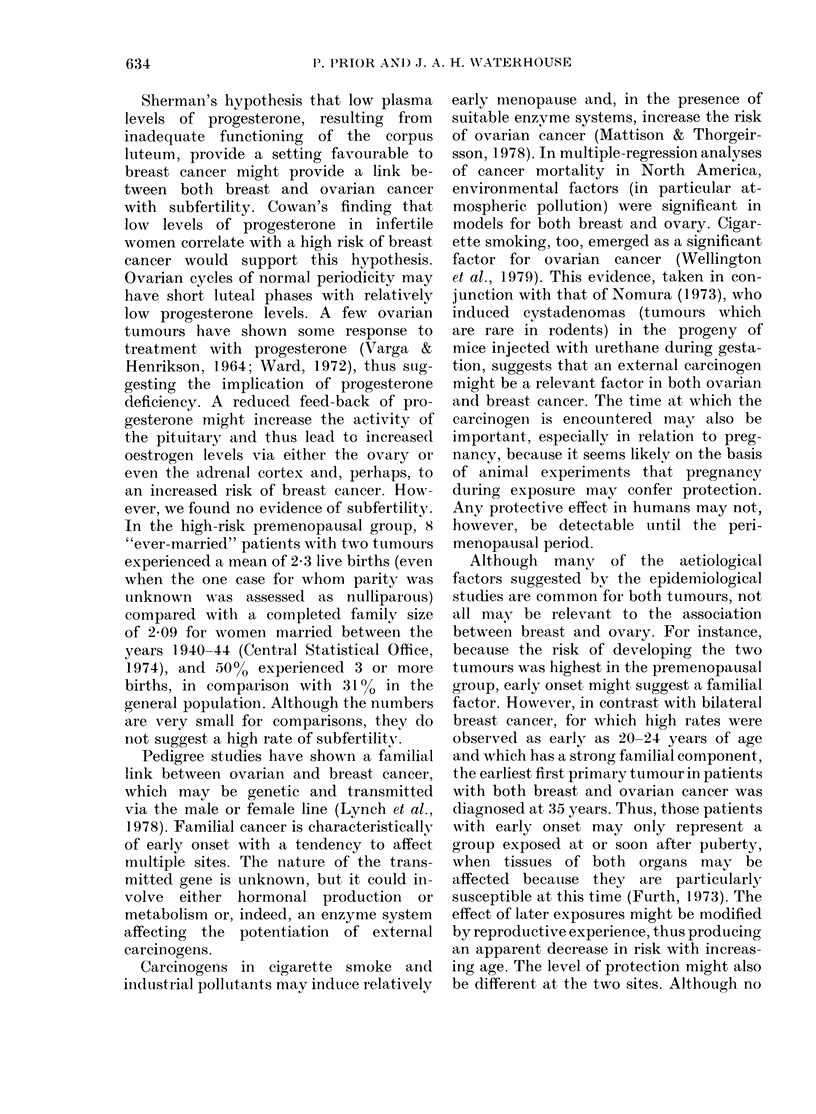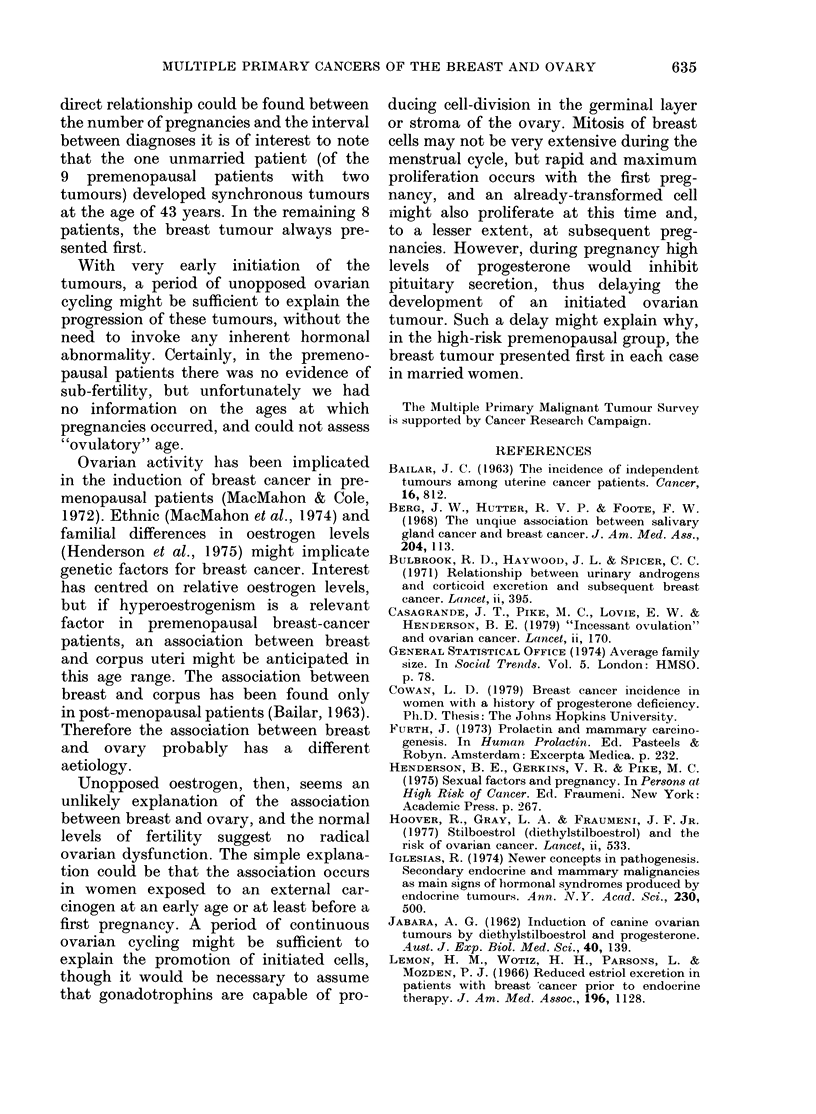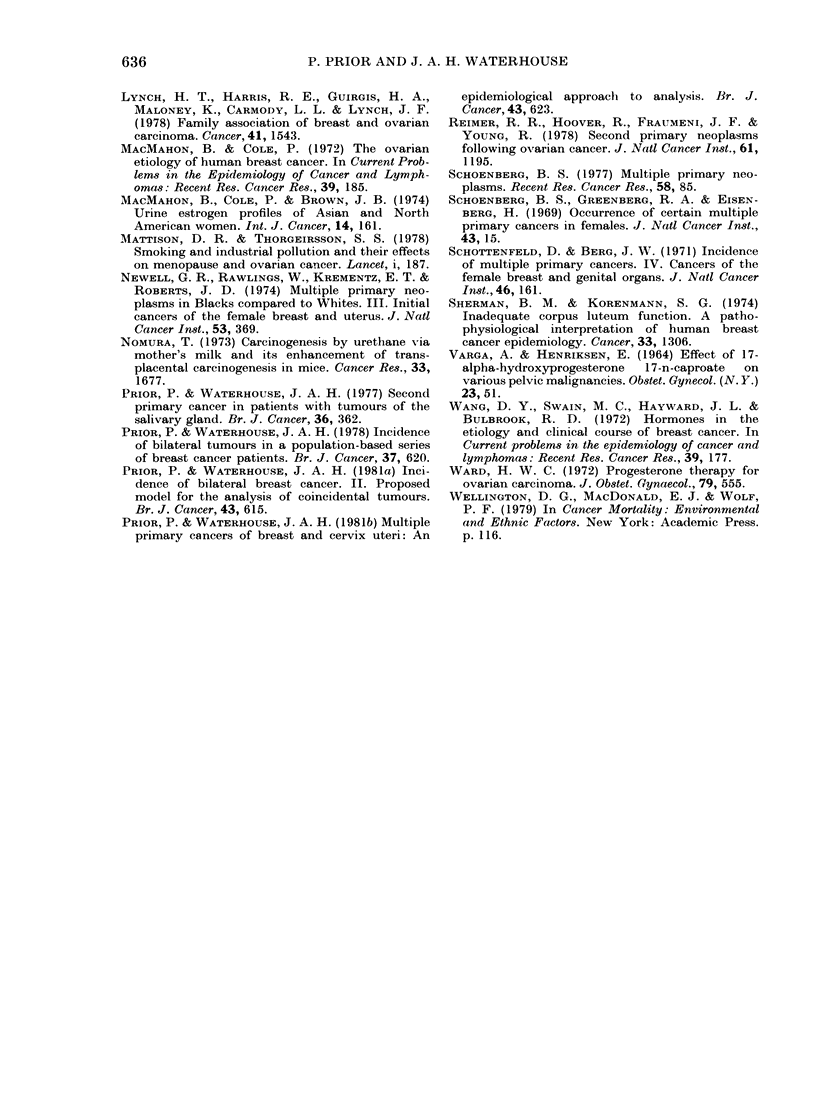# Multiple primary cancers of the breast and ovary.

**DOI:** 10.1038/bjc.1981.247

**Published:** 1981-11

**Authors:** P. Prior, J. A. Waterhouse

## Abstract

Multiple primary cancer of the breast and ovary were investigated as part of a survey being undertaken at the Birmingham and West Midlands Regional Cancer Registry. Population-based data relating to 17,756 registrations for breast and 3470 for ovarian cancer between 1950 and 1964 were analysed. On the basis of person-years at risk and incidence rates for the Region (1960-1962), an increased risk of a second primary tumour in the ovary was observed in patients diagnosed with a first primary in the breast before 45 years of age (O = 8; E = 1.83; P less than 0.001). No excess was found in patients diagnosed after 45 years of age (O = 15; E = 17.06). In patients with an index tumour of the ovary, the observed number of second primary tumours of the breast was not significantly different from the expected number (O = 19; E = 12.95). Complementary analysis (a combined assessment for the 2 sites) showed that the development of a first primary at either site before 45 years of age carried a 2.8-fold risk of a second primary tumour at the other site (O = 9; E = 3.21; P less than 0.01). After 45 years of age no increased risk was found (O = 32; E = 28.63). Over all ages a 1.3-fold risk was observed (O = 42; E = 31.54; P less than 0.05). No evidence of subfertility was found in the 9 patients in the high-risk premenopausal group who developed the 2 tumours. The results are more consistent with an aetiology of early exposure to an external carcinogen than with one of abnormal hormone production.


*Br. J. Cancer* (1981) **44,** 628

### MULTIPLE PRIMARY CANCERS OF THE BREAST AND OVARY

P. PRIOR AND J. A. H. WATERHOUSE

*From the Cancer Epidemiology Research Unit, Department of Social Medicine, University of Birmingham, England*

Received 1 April 1981 Accepted 22 July 1981

**Summary.—Multiple primary cancers of the breast and ovary were investigated as part of a survey being undertaken at the Birmingham and West Midlands Regional Cancer Registry. Population-based data relating to 17,756 registrations for breast and 3470 for ovarian cancer between 1950 and 1964 were analysed. On the basis of person-years at risk and incidence rates for the Region (1960–1962), an increased risk of a second primary tumour in the ovary was observed in patients diagnosed with a first primary in the breast before 45 years of age (O = 8; E = 1·83; *P* < 0·001). No excess was found in patients diagnosed after 45 years of age (O = 15; E = 17·06). In patients with an index tumour of the ovary, the observed number of second primary tumours of the breast was not significantly different from the expected number (O = 19; E = 12·95).**

**Complementary analysis (a combined assessment for the 2 sites) showed that the development of a first primary at either site before 45 years of age carried a 2·8-fold risk of a second primary tumour at the other site (O = 9; E = 3·21; *P* < 0·01). After 45 years of age no increased risk was found (O = 32; E = 28·63). Over all ages a 1·3-fold risk was observed (O = 42; E = 31·54; *P* < 0·05).**

**No evidence of subfertility was found in the 9 patients in the high-risk premenopausal group who developed the 2 tumours. The results are more consistent with an aetiology of early exposure to an external carcinogen than with one of abnormal hormone production.**

A SURVEY of data held at the Birmingham Regional Cancer Registry is being undertaken to establish the incidence of multiple primary cancers in the West Midlands Region. A previous paper reported the incidence of bilateral breast cancer in the Region (Prior & Waterhouse, 1978) and indicated that such tumours occurred at a higher rate in patients diagnosed with a first primary in the premenopausal period. Many independent investigations suggest the implication of endocrine hormones in the pathogenesis of breast cancer as important factors in, at least, promotion if not in initiation.

It does not seem unreasonable to suggest then that other hormone-dependent tissues might also be at risk for cancer in a patient with cancer of breast and that such a risk might be revealed by the occurrence of tumours at associated sites in the same individual. Because of the close relationship between ovarian activity and the development and metabolism of breast tissue, the incidence of tumours of ovary and breast, occurring as multiple primaries, has been examined.

*Previous studies*

Previous reports on the association between tumours of the breast and ovary are summarized in Table I. Only those surveys based on relatively large data sources and using authenticated incidence rates for computing expected numbers of tumours have been included. When breast occurred as the first primary, 3 of the 5 analyses showed a significant excess of second primaries in the ovary, RR being about 2-fold. The fifth survey (Newell

TABLE I.—*Summary of reports for breast and ovary cancer*

| Author | 1st Primary | 2nd Primary | O | E | O/E | Data source | Incidence rates |
|---|---|---|---|---|---|---|---|
| Schoenberg *et al.* (1969) | Breast | Ovary | 30 | 15·9 | 1·9** | C.T.R. | C.T.R. |
| Schottenfeld & Berg (1971) | Breast | Ovary | 24 | 11·4 | 2·1*** | M.S.C.C. | N.Y.S. |
| Newell *et al.* (1974) | Breast (Whites) | Ovary | 5 | 2·3 | 2·2 | C.H. | T.C.S. |
| Newell *et al.* (1974) | Breast (Blacks) | Ovary | 2 | 1·9 | 1·1 | C.H. | T.C.S. |
| Schoenberg (1977) | Breast | Ovary | 60 | 35·1 | 1·7*** | C.T.R. | C.T.R. |
| Schottenfeld & Berg (1971) | Ovary | Breast | 7 | 1·6 | 4·4** | M.S.C.C. | N.Y.S. |
| Schoenberg (1977) | Ovary | Breast | 25 | 17·9 | 1·4 | C.T.R. | C.T.R. |
| Reimer *et al.* (1978) | Ovary | Breast | 83 | 77·1 | 1·1 | E.R.P. | C.T.R. |

C.H. = Charity Hospital Tumor Registry, Louisiana; C.T.R. = Connecticut Tumor Registry; M.S.C.C. = Memorial Sloan–Kettering Cancer Center; N.Y.S. = New York State; T.C.S. = Ten Cities Survey; E.R.P. = End Results Program, N.C.I.
$^{**}P < 0{\cdot}01$; $^{***}P < 0{\cdot}001$.

*et al.*, 1974) suggested that there might be a differential risk between "whites" and "blacks", but the numbers involved were probably too small for any firm conclusion. Of the 3 reports available for the reverse sequence of tumours (ovary followed by breast) one indicated a significant excess of second primary tumours in the breast, RR being estimated as 4·4-fold from the hospital-based data of the Memorial Sloan-Kettering Cancer Centre, whereas the 1·4-fold risk found in the population-based series from the Connecticut Tumor Registry did not reach the 5% significance level. No significant excess was found in the series from the End Results Program data.

### MATERIALS AND METHODS

Information held at the Birmingham Regional Cancer Registry relates to a well-defined geographical and administrative region comprising 5 counties with a total population of 5 million. Covering both urban and rural communities, it is representative of the whole country. Data for the present analyses included all registrations for cancer of the breast and ovary between the years 1950 and 1964, all being followed to the 1965 anniversary of the date of first treatment, or to death if this occurred earlier. Age distributions within the 2 index sites are given in Table II.

In the first instance, the conventional approach to analysis was used: taking each index site in turn, only subsequent tumours at the other site were considered. This approach is referred to below as "Sequence"

TABLE II.—*Age distributions by index site*

| Age at diagnosis | Breast No. | Breast (%) | Ovary No. | Ovary (%) |
|---|---|---|---|---|
| 15–44 | 2899 | (16·3) | 564 | (16·3) |
| 45–59 | 6549 | (36·9) | 1462 | (42·1) |
| 60+ | 8308 | (46·8) | 1444 | (41·6) |
| Total | 17756 | (100) | 3470 | (100) |

analysis and involves the following steps. For each index site, the survival experienced by each patient was computed and entered into an array of "person-years" at risk in terms of age at and interval from the diagnosis of the first primary. Age-specific incidence rates for cancer of the second primary site were computed from registrations for the Birmingham Region between the years 1960 and 1962, together with the appropriate population figures for the region obtained from the Registrar General's Population Census in 1961. The number of second primary tumours that might be expected to occur during the period of observation were computed by applying the age-specific rates to the appropriate elements of the array of "person-years" at risk. To allow for the inclusion of coincidental diagnosis of tumours, the expected numbers were adjusted by the method described elsewhere (Prior & Waterhouse, 1981*a*), involving a model in which the parameters are the duration of a preclinical period and of clinical surveillance. In this context, "preclinical" tumours would be those that might be discovered by routine examination. For example, small non-symptomatic tumours of the breast might be discovered during a preoperative examination for an ovarian tumour, whereas a tumour of the ovary might only be found in the breast cancer patient if it carried symptoms. Thus, the preclinical period for ovarian tumours was taken as the median duration of symptoms, that is 2 months, while for breast a period of 16 months, used in previous analyses, was used.

Finally, complementary analysis (Prior & Waterhouse, 1981*b*), which combines information from the sequence analyses, was carried out. This approach attempts to reduce any methodological bias that might be inherent in the sequence analyses.

Although the primary nature of second tumours is generally assessed before registration, metastases from either breast or ovary are not uncommon at the other site. All cases were, therefore, reviewed before being included in the observed numbers of the analyses.

The significance of the differences between observed and expected numbers was assessed using the Poisson distribution.

### RESULTS

Tracing to the requisite dates was achieved for 99·5% of the breast series, yielding 62,502·5 person-years at risk for analysis. For the ovarian series tracing reached 99·9% with a yield of 7485·5 person-years.

Forty-two patients were diagnosed with primary tumours at both sites. In 6 cases the tumours were considered to be coincidental diagnoses; that is they were diagnosed on the same day or within one month of the other primary. Twenty-two breast-cancer patients subsequently developed a tumour of the ovary and 14 patients with ovarian cancer developed a tumour of the breast after an interval of more than one month.

### *Sequence analysis*

From the models it was computed that 7·94 coincidental diagnoses might be expected, 1·29 attributed to the breast series and 6·65 to the ovarian series. To preserve the integer notation for observed cases, the 6 observed coincidental cases were distributed as 1 to the breast and 5 to the ovarian series.

The results in terms of age at first primary diagnosis are summarized for the two series in Table III for 3 main age ranges, which in the absence of precise data were selected to reflect menopausal status. In the context of this analysis the results suggest that only those women who were diagnosed with a breast tumour before the age of 45 years were at an increased risk of a subsequent tumour in the ovary. In this group the excess of ovarian tumours was highly significant ($P < 0{\cdot}001$). In women diagnosed with ovarian cancer after the age of 45 years, the small excess of subsequent breast tumours did not reach statistical significance. Over all ages neither series showed a significant excess of subsequent tumours.

TABLE III.—*Sequence analysis: Observed and expected numbers of second primary tumours by index site*

| Index site | Second primary site | Age at 1st primary (years) | No. 2nd primary tumours E | O | O/E | *P* |
|---|---|---|---|---|---|---|
| Breast | Ovary | 15–44 | 1·83 | 8 | 4·4 | < 0·001 |
| | | 45–59 | 8 | 11 | 1·4 | |
| | | 60+ | 9·06 | 4 | 0·4 | |
| | | Total | 18·89 | 23 | 1·2 | |
| Ovary | Breast | 15–44 | 1·37 | 1 | 0·7 | |
| | | 45–59 | 5·64 | 9 | 1·6 | |
| | | 60+ | 5·94 | 9 | 1·5 | |
| | | Total | 12·95 | 19 | 1·5 | |

E = expected number; O = observed number.

### *Complementary analysis*

*Age at first primary diagnosis.*—When the two series were combined, the overall excess of ~10 tumours was of borderline

TABLE IV. — *Complementary analysis: Breast and ovary. Observed and expected numbers of second primary tumours*

| Age at 1st primary (years) | No. 2nd primary tumours E | O | O/E | *P* |
|---|---|---|---|---|
| 15–44 | 3·21 | 9 | 2·80 | < 0·01 |
| 45–59 | 13·63 | 20 | 1·47 | |
| 60+ | 15 | 13 | 0·87 | |
| Total | 31·84 | 42 | 1·32 | < 0·05 |

FIG. 1.—Complementary analysis of breast and ovary: observed (——) and expected (----) numbers of second primary tumours in relation to age at second primary diagnosis.

FIG. 2.—Complementary analysis of breast and ovary: cumulative observed (——) and expected (----) numbers of tumours in the breast or ovary in relation to the interval from diagnosis of the first primary. A, Ages 15–44. B, Ages 45+.

significance ($P = 0{\cdot}049$). In women with a first primary diagnosed before the age of 45 years (premenopausal group) the excess of second primary tumours was highly significant ($P < 0{\cdot}01$), whereas neither the excess in the perimenopausal group (45–59 years) nor the small deficit in the post-menopausal group (60+ years) reached statistical significance (Table IV). On combining the latter two groups (45+ years) a small excess of 4 tumours was found.

*Age at second primary diagnosis.*—Complementary analysis shows that the excess of second primary tumours occurs before the age of 60 years. After this age, the observed numbers were found to fluctuate about the expected numbers (Fig. 1).

*Interval between diagnoses.*—There was a 2·8-fold risk in the premenopausal group, and despite the small numbers the cumulative RR remained remarkably constant over time. Although RR was somewhat lower in the first year (2·4), over the remaining years the cumulative risk was about 3-fold, varying from 2·7 to 3·2.

For those patients aged 45 and over, the cumulative observed and expected numbers were close over the whole period and, although a small excess was apparent, the overall RR (1·2) was not significantly different from 1·0 (Fig. 2).

*Details of the 42 cases with 2 primary tumours*

*Histology.*—Table V shows the distribution of the histological types of ovarian tumours. For the 9 patients developing a first primary in the premenopausal period, 7 were recorded as having papillary serous cystadenocarcinoma and 2 adenocarcinoma of the ovary. The breast tumours in the patients included carcinoma (1), carcinoma in association with fibrocystic

TABLE V.—*Histological type of ovarian cancer in 42 patients with 2 primary tumours*

| Histological type | Pre-menopausal | Peri-menopausal | Post-menopausal | All ages (% of total) |
|---|---|---|---|---|
| | Age range at 1st primary diagnosis No. of patients (% of age range) | | | |
| Papillary serous cystadenocarcinoma; adenocarcinoma | 9 (100) | 13 (65) | 6 (46·1) | 28 (66·7) |
| Pseudomucinous carcinoma | — | 3 (15) | 4 (30·8) | 7 (16·6) |
| Granulosa cell carcinoma | — | — | 2 (15·4) | 2 (4·8) |
| Teratoma | — | 2 (10) | — | 2 (4·8) |
| Not known | — | 2 (10) | 1 (7·7) | 3 (7·1) |
| Total (% of total) | 9 (21·4) | 20 (47·6) | 13 (31) | 42 (100) |

disease (2), spheroidal-cell carcinoma (3), anaplastic (2) and undifferentiated carcinoma (1). The last patient did, however, survive to develop a medullary carcinoma of the opposite breast, and an adenocarcinoma of the caecum as a fourth primary. Pseudomucinous adenocarcinoma of the ovary was recorded only in the peri- and postmenopausal groups. Teratomas were diagnosed in 2 perimenopausal patients, and 2 granulosa-cell tumours were found in patients over 60 at first primary.

*Marital status and parity.*—Marital status was unknown in only one of the patients with the 2 primary tumours. Although the numbers are small the proportion of single women (11·9%) was similar to that in the general population (11·7%) for a group of women aged 55–59. The mean age at first primary diagnosis was 56 years for the 42 observed cases in the survey.

TABLE VI.—*Menopausal status at 1st primary diagnosis and parity of 42 patients with 2 primary tumours*

| Parity | Marital status: Ever-married: Pre-menopausal | Other | All | Single | Not known |
|---|---|---|---|---|---|
| 0 | 0 | 2 | 2 | | |
| 1 | 1 | 5 | 6 | | |
| 2 | 3 | 2 | 5 | | |
| 3 | 1 | — | 1 | | |
| 4 | 2 | 1 | 3 | | |
| 5 | 0 | 1 | 1 | | |
| Not known | 1 | 17 | 18 | | |
| Total | 8 | 28 | 36 (85·7%) | 5 (11·9) | 1 (2·4%) |

Information on parity was sparse for patients over the age of 45 but in the premenopausal group of "ever-married" patients, the information was missing in only one out of 8 cases. Even assuming that this case was non-parous, the mean numbers of births was 2·3 (Table VI).

### DISCUSSION

When breast was taken as the index site, sequence analysis indicated a strong association between tumours of breast and ovary in the premenopausal group, and an apparent decrease in RR with increasing age at first primary diagnosis. For the reverse sequence of tumours, the association was not so clear-cut. Small excesses or deficits of observed tumours may arise from spurious divisions in the population under consideration, and may therefore be the effect of methodology rather than aetiology. Complementary analysis attempts to make some allowance for effects arising from methodology and, in this instance, while complementary analysis supports the association in the premenopausal group, with a more conservative estimate of RR, it indicates that for the remaining patients the observed number of tumours (33) was close to the expected number (28·63). These results are strongly suggestive that menopausal status is important to the association. The raised, but non-significant, risk in the perimenopausal patients may therefore be due to heterogeneity of this group with respect to status.

Although problems in differential diagnosis might have been anticipated for these two sites, complementary analysis indicated that the cumulative RR in the premenopausal group was constant over the period of observation, at least after the first 2 years. RR was somewhat lower for these 2 years, which suggests that we have been over-cautious in accepting the presence of a second primary, probably when one or both primaries presented at a late stage.

Because of the different methods of analysis, it is difficult to compare the results from the Birmingham data with other published reports. However, for two series—Memorial Hospital, New York (M.S.C.C.) and Connecticut Tumor Registry (C.T.R.)—the results of sequence analyses for pairs of sites can be combined to give an approximate parallel to complementary analysis. The overall RR for *breast with ovary* then becomes 2·48 (O = 31; E = 13·0) for M.S.C.C. and 1·6 (O = 85; E = 53·0) for C.T.R., in comparison with 1·3 for the Birmingham data. The result for M.S.C.C. suggests that the hospital population from which the series was drawn was highly selected for younger women, an inference that was drawn (Prior & Waterhouse, 1977) from a previous report from this centre (Berg *et al.*, 1968). The small difference between C.T.R. and Birmingham could be due, in part, to the statistical treatment of coincidental tumours, as well as to a differing age distribution at first primary diagnosis.

An association between functionally related sites such as breast and ovary suggests that hormonal influences should at least be considered as an explanation of the relationship (Schoenberg, 1977). The finding that the increased risk was confined almost entirely to premenopausal patients supports this view. Although the role of hormones in the aetiology of breast cancer is still far from clear, oestrogens are regarded by some as the prime suspect, mainly on the basis of animal experiments and because some human tumours respond to hormone therapy. Others favour a theory of abnormal androgen metabolism (Bulbrook *et al.*, 1971) while progesterone-deficiency as a risk factor also has its advocates (Sherman and Korenmann, 1974; Cowan, 1979). Epidemiological factors such as marital status, reproductive history and early castration have been found to be associated with the risk of breast cancer, thereby suggesting that ovarian activity, whether directly related to hormones or not, plays some part in tumour development. It seems unlikely that a unified theory could account for the whole spectrum of breast cancer, even one as non-specific as "ovarian activity", unless any abnormality in the endocrine feed-back system represents a risk factor for hormone-dependent tissues. Steroid abnormalities may be multi-directional, and relative proportions may be more important than the absolute level of an individual hormone (Lemon *et al.*, 1966; Wang *et al.*, 1972).

Single status and low parity have also been suggested as high-risk factors for ovarian cancer. "Ovulatory age", that is the number of ovarian cycles experienced, has been shown to correlate directly with risk (Casagrande *et al.*, 1979). We found no evidence to support an excess of single women among those developing the two tumours.

Although ovarian tumours have been induced in animals by oestrogens (Jabara, 1962), oestrogens have only recently been implicated in the human disease (Hoover *et al.*, 1977). In rats, implanted pituitary tumours, secreting LH and FSH, induce oestrogen-secreting granulosa-cell tumours of the ovary which, on transplantation to a new host, induce mammary tumours (Iglesias, 1974). Pituitary stimulation may be important in the human situation with respect to ovarian cancers, but only 3–4% of human tumours are of the granulosa-cell type, more than 80% being cystadenocarcinoma or solid adenocarcinomas, which are not usually hormone secreting and, therefore, unlikely to be responsible for the association with breast cancer demonstrated here.

Sherman's hypothesis that low plasma levels of progesterone, resulting from inadequate functioning of the corpus luteum, provide a setting favourable to breast cancer might provide a link between both breast and ovarian cancer with subfertility. Cowan's finding that low levels of progesterone in infertile women correlate with a high risk of breast cancer would support this hypothesis. Ovarian cycles of normal periodicity may have short luteal phases with relatively low progesterone levels. A few ovarian tumours have shown some response to treatment with progesterone (Varga & Henrikson, 1964; Ward, 1972), thus suggesting the implication of progesterone deficiency. A reduced feed-back of progesterone might increase the activity of the pituitary and thus lead to increased oestrogen levels via either the ovary or even the adrenal cortex and, perhaps, to an increased risk of breast cancer. However, we found no evidence of subfertility. In the high-risk premenopausal group, 8 "ever-married" patients with two tumours experienced a mean of 2·3 live births (even when the one case for whom parity was unknown was assessed as nulliparous) compared with a completed family size of 2·09 for women married between the years 1940–44 (Central Statistical Office, 1974), and 50% experienced 3 or more births, in comparison with 31% in the general population. Although the numbers are very small for comparisons, they do not suggest a high rate of subfertility.

Pedigree studies have shown a familial link between ovarian and breast cancer, which may be genetic and transmitted via the male or female line (Lynch *et al.*, 1978). Familial cancer is characteristically of early onset with a tendency to affect multiple sites. The nature of the transmitted gene is unknown, but it could involve either hormonal production or metabolism or, indeed, an enzyme system affecting the potentiation of external carcinogens.

Carcinogens in cigarette smoke and industrial pollutants may induce relatively early menopause and, in the presence of suitable enzyme systems, increase the risk of ovarian cancer (Mattison & Thorgeirsson, 1978). In multiple-regression analyses of cancer mortality in North America, environmental factors (in particular atmospheric pollution) were significant in models for both breast and ovary. Cigarette smoking, too, emerged as a significant factor for ovarian cancer (Wellington *et al.*, 1979). This evidence, taken in conjunction with that of Nomura (1973), who induced cystadenomas (tumours which are rare in rodents) in the progeny of mice injected with urethane during gestation, suggests that an external carcinogen might be a relevant factor in both ovarian and breast cancer. The time at which the carcinogen is encountered may also be important, especially in relation to pregnancy, because it seems likely on the basis of animal experiments that pregnancy during exposure may confer protection. Any protective effect in humans may not, however, be detectable until the perimenopausal period.

Although many of the aetiological factors suggested by the epidemiological studies are common for both tumours, not all may be relevant to the association between breast and ovary. For instance, because the risk of developing the two tumours was highest in the premenopausal group, early onset might suggest a familial factor. However, in contrast with bilateral breast cancer, for which high rates were observed as early as 20–24 years of age and which has a strong familial component, the earliest first primary tumour in patients with both breast and ovarian cancer was diagnosed at 35 years. Thus, those patients with early onset may only represent a group exposed at or soon after puberty, when tissues of both organs may be affected because they are particularly susceptible at this time (Furth, 1973). The effect of later exposures might be modified by reproductive experience, thus producing an apparent decrease in risk with increasing age. The level of protection might also be different at the two sites. Although no direct relationship could be found between the number of pregnancies and the interval between diagnoses it is of interest to note that the one unmarried patient (of the 9 premenopausal patients with two tumours) developed synchronous tumours at the age of 43 years. In the remaining 8 patients, the breast tumour always presented first.

With very early initiation of the tumours, a period of unopposed ovarian cycling might be sufficient to explain the progression of these tumours, without the need to invoke any inherent hormonal abnormality. Certainly, in the premenopausal patients there was no evidence of sub-fertility, but unfortunately we had no information on the ages at which pregnancies occurred, and could not assess "ovulatory" age.

Ovarian activity has been implicated in the induction of breast cancer in premenopausal patients (MacMahon & Cole, 1972). Ethnic (MacMahon *et al.*, 1974) and familial differences in oestrogen levels (Henderson *et al.*, 1975) might implicate genetic factors for breast cancer. Interest has centred on relative oestrogen levels, but if hyperoestrogenism is a relevant factor in premenopausal breast-cancer patients, an association between breast and corpus uteri might be anticipated in this age range. The association between breast and corpus has been found only in post-menopausal patients (Bailar, 1963). Therefore the association between breast and ovary probably has a different aetiology.

Unopposed oestrogen, then, seems an unlikely explanation of the association between breast and ovary, and the normal levels of fertility suggest no radical ovarian dysfunction. The simple explanation could be that the association occurs in women exposed to an external carcinogen at an early age or at least before a first pregnancy. A period of continuous ovarian cycling might be sufficient to explain the promotion of initiated cells, though it would be necessary to assume that gonadotrophins are capable of producing cell-division in the germinal layer or stroma of the ovary. Mitosis of breast cells may not be very extensive during the menstrual cycle, but rapid and maximum proliferation occurs with the first pregnancy, and an already-transformed cell might also proliferate at this time and, to a lesser extent, at subsequent pregnancies. However, during pregnancy high levels of progesterone would inhibit pituitary secretion, thus delaying the development of an initiated ovarian tumour. Such a delay might explain why, in the high-risk premenopausal group, the breast tumour presented first in each case in married women.

The Multiple Primary Malignant Tumour Survey is supported by Cancer Research Campaign.

REFERENCES

BAILAR, J. C. (1963) The incidence of independent tumours among uterine cancer patients. *Cancer*, **16,** 812.

BERG, J. W., HUTTER, R. V. P. & FOOTE, F. W. (1968) The unqiue association between salivary gland cancer and breast cancer. *J. Am. Med. Ass.*, **204,** 113.

BULBROOK, R. D., HAYWOOD, J. L. & SPICER, C. C. (1971) Relationship between urinary androgens and corticoid excretion and subsequent breast cancer. *Lancet*, ii, 395.

CASAGRANDE, J. T., PIKE, M. C., LOVIE, E. W. & HENDERSON, B. E. (1979) "Incessant ovulation" and ovarian cancer. *Lancet*, ii, 170.

GENERAL STATISTICAL OFFICE (1974) Average family size. In *Social Trends*. Vol. 5. London: HMSO. p. 78.

COWAN, L. D. (1979) Breast cancer incidence in women with a history of progesterone deficiency. Ph.D. Thesis: The Johns Hopkins University.

FURTH, J. (1973) Prolactin and mammary carcinogenesis. In *Human Prolactin*. Ed. Pasteels & Robyn. Amsterdam: Excerpta Medica. p. 232.

HENDERSON, B. E., GERKINS, V. R. & PIKE, M. C. (1975) Sexual factors and pregnancy. In *Persons at High Risk of Cancer*. Ed. Fraumeni. New York: Academic Press. p. 267.

HOOVER, R., GRAY, L. A. & FRAUMENI, J. F. JR. (1977) Stilboestrol (diethylstilboestrol) and the risk of ovarian cancer. *Lancet*, ii, 533.

IGLESIAS, R. (1974) Newer concepts in pathogenesis. Secondary endocrine and mammary malignancies as main signs of hormonal syndromes produced by endocrine tumours. *Ann. N.Y. Acad. Sci.*, **230,** 500.

JABARA, A. G. (1962) Induction of canine ovarian tumours by diethylstilboestrol and progesterone. *Aust. J. Exp. Biol. Med. Sci.*, **40,** 139.

LEMON, H. M., WOTIZ, H. H., PARSONS, L. & MOZDEN, P. J. (1966) Reduced estriol excretion in patients with breast cancer prior to endocrine therapy. *J. Am. Med. Assoc.*, **196,** 1128.

LYNCH, H. T., HARRIS, R. E., GUIRGIS, H. A., MALONEY, K., CARMODY, L. L. & LYNCH, J. F. (1978) Family association of breast and ovarian carcinoma. *Cancer*, **41**, 1543.

MACMAHON, B. & COLE, P. (1972) The ovarian etiology of human breast cancer. In *Current Problems in the Epidemiology of Cancer and Lymphomas: Recent Res. Cancer Res.*, **39**, 185.

MACMAHON, B., COLE, P. & BROWN, J. B. (1974) Urine estrogen profiles of Asian and North American women. *Int. J. Cancer*, **14**, 161.

MATTISON, D. R. & THORGEIRSSON, S. S. (1978) Smoking and industrial pollution and their effects on menopause and ovarian cancer. *Lancet*, i, 187.

NEWELL, G. R., RAWLINGS, W., KREMENTZ, E. T. & ROBERTS, J. D. (1974) Multiple primary neoplasms in Blacks compared to Whites. III. Initial cancers of the female breast and uterus. *J. Natl Cancer Inst.*, **53**, 369.

NOMURA, T. (1973) Carcinogenesis by urethane via mother's milk and its enhancement of transplacental carcinogenesis in mice. *Cancer Res.*, **33**, 1677.

PRIOR, P. & WATERHOUSE, J. A. H. (1977) Second primary cancer in patients with tumours of the salivary gland. *Br. J. Cancer*, **36**, 362.

PRIOR, P. & WATERHOUSE, J. A. H. (1978) Incidence of bilateral tumours in a population-based series of breast cancer patients. *Br. J. Cancer*, **37**, 620.

PRIOR, P. & WATERHOUSE, J. A. H. (1981*a*) Incidence of bilateral breast cancer. II. Proposed model for the analysis of coincidental tumours. *Br. J. Cancer*, **43**, 615.

PRIOR, P. & WATERHOUSE, J. A. H. (1981*b*) Multiple primary cancers of breast and cervix uteri: An epidemiological approach to analysis. *Br. J. Cancer*, **43**, 623.

REIMER, R. R., HOOVER, R., FRAUMENI, J. F. & YOUNG, R. (1978) Second primary neoplasms following ovarian cancer. *J. Natl Cancer Inst.*, **61**, 1195.

SCHOENBERG, B. S. (1977) Multiple primary neoplasms. *Recent Res. Cancer Res.*, **58**, 85.

SCHOENBERG, B. S., GREENBERG, R. A. & EISENBERG, H. (1969) Occurrence of certain multiple primary cancers in females. *J. Natl Cancer Inst.*, **43**, 15.

SCHOTTENFELD, D. & BERG, J. W. (1971) Incidence of multiple primary cancers. IV. Cancers of the female breast and genital organs. *J. Natl Cancer Inst.*, **46**, 161.

SHERMAN, B. M. & KORENMANN, S. G. (1974) Inadequate corpus luteum function. A pathophysiological interpretation of human breast cancer epidemiology. *Cancer*, **33**, 1306.

VARGA, A. & HENRIKSEN, E. (1964) Effect of 17-alpha-hydroxyprogesterone 17-n-caproate on various pelvic malignancies. *Obstet. Gynecol.* (*N.Y.*) **23**, 51.

WANG, D. Y., SWAIN, M. C., HAYWARD, J. L. & BULBROOK, R. D. (1972) Hormones in the etiology and clinical course of breast cancer. In *Current problems in the epidemiology of cancer and lymphomas: Recent Res. Cancer Res.*, **39**, 177.

WARD, H. W. C. (1972) Progesterone therapy for ovarian carcinoma. *J. Obstet. Gynaecol.*, **79**, 555.

WELLINGTON, D. G., MACDONALD, E. J. & WOLF, P. F. (1979) In *Cancer Mortality: Environmental and Ethnic Factors*. New York: Academic Press. p. 116.